# Standardized 3D Transoesophageal Echocardiography Manoeuvre for Enhanced Tenting Height Evaluation During Transcatheter Mitral Valve Edge-to-Edge Repair

**DOI:** 10.3390/jcm13216525

**Published:** 2024-10-30

**Authors:** Michela Bonanni, Giancarlo Trimarchi, Giovanni Benedetti, Andreina D’Agostino, Giuseppe Iuliano, Rachele Manzo, Rosangela Capasso, Elisa Cerone, Umberto Paradossi, Sergio Berti, Massimiliano Mariani

**Affiliations:** 1 Fondazione Toscana G. Monasterio, Ospedale del Cuore G. Pasquinucci, 54100 Massa, Italy; giovanni.benedetti@ftgm.it (G.B.); adagostino@monasterio.it (A.D.); cerone@monasterio.it (E.C.); ifcberti@ftgm.it (S.B.); marianims@ftgm.it (M.M.); 2 Department of Experimental Medicine, University of Rome Tor Vergata, 00133 Rome, Italy; 3 Department of Clinical and Experimental Medicine, University of Messina, 98100 Messina, Italy; giancarlo.trimarchi18@gmail.com; 4 Interdisciplinary Center for Health Sciences, Scuola Superiore Sant’Anna, 56127 Pisa, Italy; 5 Cardiovascular Department, University Hospital San Giovanni di Dio e Ruggi d’Aragona, 84131 Salerno, Italy; gi.iuliamo93@gmail.com; 6 Department of Advanced Biomedical Sciences, Federico II University of Naples, Via S. Pansini, 80131 Naples, Italy; rachele4manzo@gmail.com; 7 Department of Clinical and Molecular Medicine, Division of Cardiology, Sapienza, University of Rome, 00185 Rome, Italy; capassorosangela@gmail.com

**Keywords:** mitral valve, transcatheter edge-to-edge repair, mitraclip, mitral regurgitation, trans-septal puncture

## Abstract

**Background:** Transcatheter treatments for structural heart disease, including edge-to-edge mitral valve repair (mTEER), heavily rely on transoesophageal echocardiography (TOE) for pre-procedural assessment and guidance. Trans-septal puncture (TSP) is one of the first key steps of such procedures, with two-dimensional (2D) TOE often providing suboptimal imaging. Three-dimensional (3D) echocardiography could overcome the limitations of 2D TOE and improve the assessment of tenting height. **Methods:** This single-centre, retrospective study included 64 patients who underwent mTEER between October 2023 and April 2024. Tenting height during TSP was assessed by 2D TOE in mid-oesophageal (ME) four-chamber view and by 3D TOE after the acquisition of a 3D volume, including the interatrial septum, aortic valve, and mitral valve, and subsequent multiplanar reconstruction (MPR). A total of 100 TSP attempts with 2D and 3D TOE were evaluated. **Results:** Procedural success was obtained in 92.2% of cases. There was a statistically significant difference between 2D and 3D measurements (2D: 4.36 ± 0.61 cm, MPR: 4.59 ± 0.63 mm^2^, *p* < 0.001), despite good correlation (r = 0.880, *p* < 0.001). The difference between 2D and 3D tenting height measurements differed significantly between patients with optimal and suboptimal 2D image windows (*p* < 0.001). There was no significant difference in septal puncture success between optimal 2D and 3D data (*p* = 0.942). **Conclusions:** Adopting 3D TOE with multiplanar reconstruction for assessing tenting height in mTEER procedures offers significant advantages over traditional 2D TOE. Enhanced visualization, accuracy, and potential for standardization improve procedural outcomes and patient safety, making 3D TOE integration into routine practice highly beneficial and recommended.

## 1. Introduction

In the last decade, transcatheter treatment strategies for structural heart disease, including edge-to-edge mitral valve repair (mTEER), have spread worldwide and today represent a leading therapeutic option in different clinical scenarios and patient settings [1]. Transoesophageal echocardiography (TOE) plays a key role in pre-procedural screening and is essential in guiding and monitoring all procedural phases of mTEER [2,3,4]. Trans-septal puncture (TSP) is the first key step during mTEER, as well as during all percutaneous treatment strategies in which direct access to the left atrium (LA) is needed, requiring optimal imaging and experienced interventionists [5,6,7]. The mid-oesophageal (ME) aortic valve short axis (SAX) view and the ME bicaval view (BC), in single-plane or biplane mode, are the main two-dimensional (2D) TOE views for the assessment of the interatrial septum (IAS) and for monitoring the tenting of the fossa ovalis (FO) [2,3]. An ME four-chamber view is usually used to monitor TSP height, which should provide enough working distance between the puncture site and mitral valve (MV) leaflet coaptation line. The ideal puncture position depends on the specific coaptation device, mitral regurgitation (MR) mechanism, and MV anatomy, but it should always be on the superior and posterior part of the FO [5]. Sometimes, the accurate identification of tenting height may be challenging due to the inability to adequately visualize the entire FO and the MV leaflet coaptation point in a single 2D TOE view [8,9]. Three-dimensional (3D) TOE could overcome the limitations of 2D echocardiography by obtaining a large 3D volume, including IAS and MV leaflets, and then reprocessing it through the multiplanar reconstruction (MPR) tool [10]. The 3D dataset can be reprocessed into multiple views, allowing the physician to explore the anatomy from different angles and obtain a more accurate representation of the tenting height and related structures. In this study, we propose a simple and standardized manoeuvre to adequately assess tenting height during TSP for mTEER using 3D TOE.

## 2. Materials and Methods

This was a single-centre, retrospective study in which data were collected from all consecutive patients with severe MV regurgitation undergoing mTEER at our institution between October 2021 and April 2024. A total of sixty-four patients were included in this study. For each patient, all different TSP attempts made during the procedure were recorded using a commercially available ultrasound system (Philips Epiq; Philips Medical Systems, Andover, MA, USA) with a matrix-array 3D-TEE probe (X7–2 t; Philips Medical Systems). The positioning of the TSP needle was guided by standard TOE views [3]. Once the tip of the needle had been positioned in the planned puncture site, the height of FO tenting with respect to the plane of MV coaptation was evaluated in both 2D and 3D through the MPR tool. In total, 100 TSP attempts were recorded and measured in both imaging modalities by a single experienced operator. For qualification purposes, the severity of MR was assessed according to the criteria recommended by European valvular heart disease guidelines [11]. Procedural success was defined as post-procedural MR grades of less than or equal to 2+ [12,13].

### 2.1. Two-Dimensional Method

**Figure 1:**
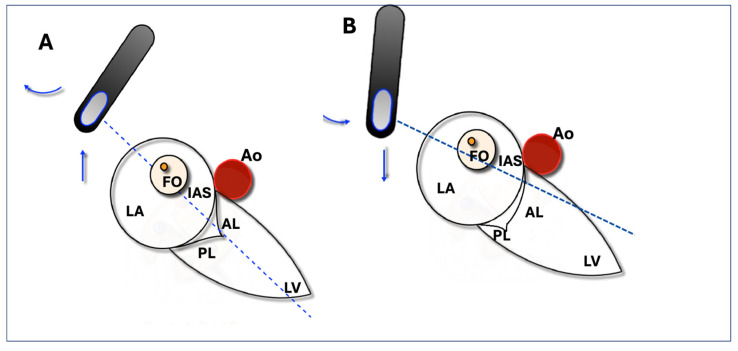
Optimal and suboptimal TOE views. (**A**) shows an accurate determination of tenting height using a two-dimensional upper oesophageal transoesophageal echocardiography view at 0–40°, visualizing the entire fossa ovalis (FO) and mitral valve leaflet coaptation point in a single view during the end-systolic phase. (**B**) shows a suboptimal view visualizing the FO with only the anterior mitral valve leaflet. AL: anterior leaflet; Ao: aortic valve, FO: fossa ovalis; IAS: interatrial septum; LA: left atrium; LV: left ventricle; PL: posterior leaflet.

An ME or upper oesophageal (UE) 4-chamber view with slight retroflection of the probe was obtained to include both the FO and the MV leaflet coaptation point. A 2D assessment of tenting height was considered adequate when tenting and the MV leaflet coaptation were clearly seen in the same plane. The view is considered suboptimal if it does not include both structures or if the aortic root is visible in the same plane (Figure 1). Even though every TSP attempt was evaluated in both imaging modalities (2D and 3D with MPR), when the 2D view was considered suboptimal, the site of puncture was chosen based on 3D data. Otherwise, it was based on 2D data.

### 2.2. MPR Method

**Figure 2:**
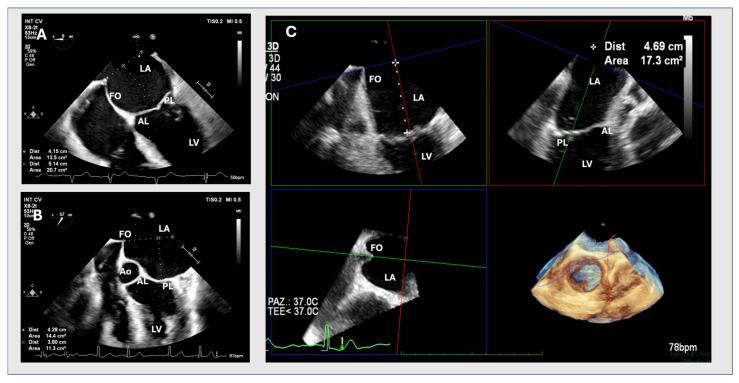
Figures (**A**,**B**) show an optimal 2D and suboptimal upper transoesophageal view, respectively. Figure (**C**) shows a multiplanar three-dimensional reconstruction. AL: anterior leaflet; Ao: aortic valve, FO: fossa ovalis; LA: left atrium; LV: left ventricle; PL: posterior leaflet.

In the 3D method, a full volume including the IAS, MV, and aortic valve is obtained. The volume is then analysed using the MPR tool with all planes fixed perpendicular to each other. The centre of the three axes is positioned in the middle of the LA in all panels and rotated to obtain a short axis of the LA. In this plane, the axes are then adjusted so that one plane crosses the tenting point; in the other two panels, the axes are adjusted so that one plane passes through the MV coaptation point. With a step-by-step alignment of the axes, a 4-chamber view with tenting and MV leaflet coaptation is obtained in one panel. In this view, tenting height can be measured without shortening (Figure 2).

### 2.3. Statistical Analysis

**Figure 3:**
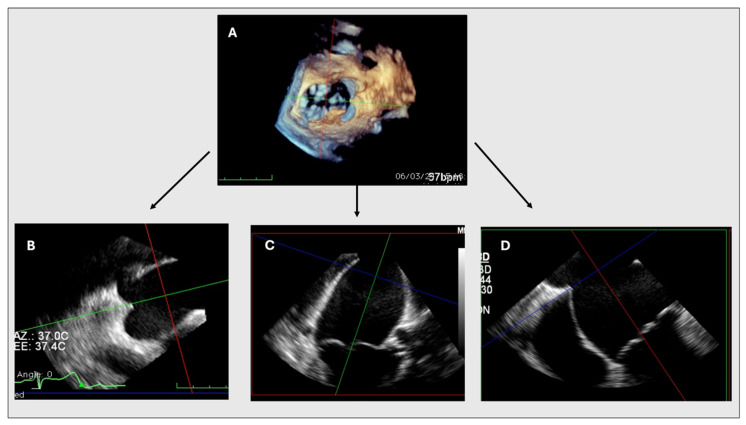
MPR. MPR allows for the reconstruction of different planes and views from the 3D dataset, including the interatrial septum, mitral valve (MV), and aortic valve (**A**). Figure (**B**) shows a short axis of the left atrium, with a sectional plane set to cross the tenting point. In figures (**C**,**D**), the longitudinal plane is set to pass through the MV coaptation point. An adequate 4-chamber view (**D**) is obtained allowing the measurement of the tenting height without shortening.

Continuous variables were expressed as mean ± standard deviation or median and inter-quartile range (IQR), according to a Gaussian or non-Gaussian distribution. Normality was tested using the Shapiro–Wilks test. Categorical variables were expressed as frequencies and percentages. The different measurements were compared in the same patient using the Student *t*-test for paired data or the Mann–Whitney test, according to data distribution. The Spearman correlation coefficient was used to assess the relationship between 2D and MPR measurements. Agreement between techniques was plotted using the Bland–Altman method (Figure 3). All computations relied on commercially available software (SPSS IBMS v21 for Mac; SPPS Inc., Chicago, IL, USA and JMPw 11; SAS Institute Inc., Cary, NC, USA), with statistical significance set at p 0.05.

## 3. Results

### 3.1. Baseline Patient Characteristics

Table 1 summarizes the clinical characteristics of the study population. During mTEER, in 12 (18.8%) patients, 2D views for TH were considered suboptimal. The mean age was 78.5 ± 8.2 years; 39% were women. The mean body mass index (BMI) was 25.4 ± 4.4. The majority of patients had hypertension (75%) and hyperlipidaemia (57.8%), 32.8% had diabetes mellitus, 31.3% had a history of myocardial infarction, and 23.4% had undergone percutaneous coronary intervention. In addition, 17.2% had undergone coronary artery bypass grafting and 7.9% of patients had already undergone heart valve surgery. Atrial fibrillation or atrial flutter was documented in 57.8% of patients, while 9.5% of patients had a cardiac resynchronization device. A significant percentage of patients (58%) were classified as New York Heart Association class ≥ III. No significant differences in baseline parameters were found between patients with optimal and suboptimal imaging.

### 3.2. Baseline MR Echocardiographic Parameters

Baseline echocardiographic parameters are listed in Table 2. The aetiology of MR was classified as primary mitral regurgitation (PMR) in 39.1% of cases and secondary mitral regurgitation (SMR) in 60.9%. Of the patients with SMR, 37.5% had ischemic SMR and 23.4% had non-ischemic SMR. The median effective regurgitant orifice area (EROA) was 0.45 cm^2^ (IQR 0.40–0.65), with a regurgitant volume (RV) of 63 mL (IQR 49–85). The 3D vena contracta area was 0.45 cm^2^ (IQR 0.35–0.7). The median MV area was 7 cm^2^ (IQR 5–7.75), with a transvalvular gradient of 2 mmHg (IQR 1–2). The median LA volume indexed by body surface area was 61 mL/mq (IQR 49, 5–73). The median left ventricular ejection fraction was 50% (IQR 30.5–60).

### 3.3. Procedural Characteristics and Echocardiographic Results

In 68.8% of patients, MR was treated with a single edge-to-edge device, in 29.7% with two devices and in 1.6% with three devices. Procedural success was obtained in 92.2% of cases. Post-procedural EROA was 0.16 cm^2^ (0.12–0.21), while RV was 21 mL (16.25–27.5). The 3D vena contracta area was 0.18 cm^2^ (0.12–0.22). Mean transvalvular gradient was 3 mmHg (3–4). No pericardial effusion or cardiac perforation occurred.

### 3.4. Correlations Between 2D and MPR Measurements

**Figure 4:**
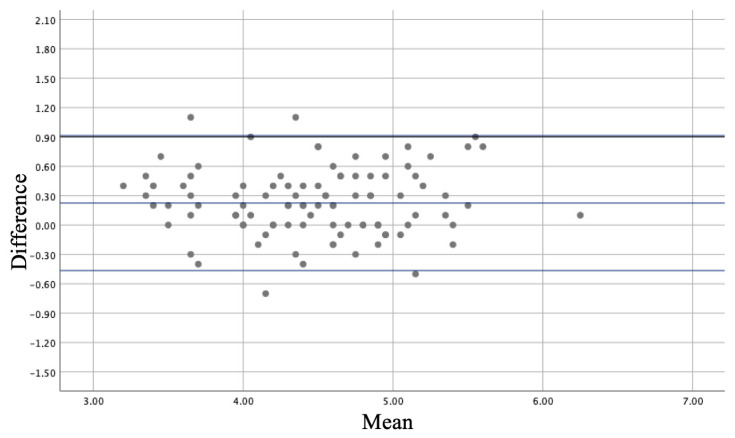
Bland–Altman plot.

Despite the good correlation between 2D and 3D tenting height measurements (r 0.880 95% CI: 0.81–0.91, *p* < 0.001), we found a statistically significant difference between those two measures (2D: 4.36 ± 0.61 cm, MPR: 4.59 ± 0.63 mm^2^, *p* < 0.001, Table 3, Table 4 and Table 5). The Bland–Altman analysis (Figure 4) assessed the different TH measurements between 2D and MPR methods. In addition, the median difference between 2D and 3D septal height measurements was statistically different in patients with optimal versus suboptimal 2D windows (0.2 vs. 0.5, *p* = 0.0001). However, the difference in procedural success between septal punctures based on 2D data with an optimal window and those based on 3D data when 2D was considered suboptimal was not statistically significant (*p* = 0.942).

## 4. Discussion

The present study proposes a simple and standardized manoeuvre for assessing tenting height during TSP using 3D TOE, which aims to improve the accuracy of the procedure. The main results are as follows: (1) there is a good correlation between the 2D TSP and MPR TSP measurements, (2) the measurements obtained by the two methods show a statistically significant difference, (3) in 18,8% of patients, the 2D view for TSP was considered suboptimal, and (4) there is no significant difference in procedural success between optimal 2D and 3D data. Accurately determining the tenting height using 2D TOE is a challenge, as it is difficult to visualize the entire FO and MV leaflet coaptation point in a single view [14]. This is especially relevant in patients with severe LA dilatation, so that 2D imaging is not able to show the full extent of the IAS in one or two image planes. Since a significant number of patients with MR also have severe LA enlargement, this means that using 2D-TOE alone may lead to a significant number of procedures in which tenting height may not be accurately assessed, potentially compromising the success of the mTEER procedure [15]. These limitations can be overcome by 3D-TOE, as it captures a large 3D volume containing the IAS and MV leaflets. This volume can then be post-processed with the MPR tool to obtain a more detailed and accurate assessment than would be possible with a single 2D view, ensuring a precise TSP during mTEER [16]. The application of MPR in TSP has already been assessed. Mahmoud et al. [17] evaluated the use of a new manoeuvre using 3DTOE to visualize IAS in 20 patients undergoing TSP, concluding that the 3D method used is feasible, reduces fluoroscopy time, and minimizes complications [16]. In addition, the use of MPR with real-time 3D guidance proved particularly important and useful in identifying the ideal position for TSP even in the presence of atrial septal occluders during M-TEEER [18]. Our study is the first to include one hundred TSP attempts and to compare the height of FO puncture between the 2D and 3D methods. The high correlation coefficient between 2D and MPR measurements suggests that although 2D measurements are close, they may not consistently capture the accurate dimensions needed to optimize treatment outcomes. Despite the good correlation, the measurements obtained using the two methods showed a statistically significant difference. Furthermore, there is a statistically significant difference in the median difference between 2D and 3D septal height measurements in patients with optimal versus suboptimal 2D windows. However, procedural success rates between 2D (optimal) and 3D (suboptimal 2D) methods were not significantly different. Those results show that the quality of 2D imaging can influence the accuracy of the measurement. Optimal 2D windows provide more reliable data compared to suboptimal windows, which can conceal anatomical details. In addition, the results indicate that 3D MPR provides a more precise (and likely more accurate) measurement of tenting height. The differences, although seemingly small, can be critical in the delicate mTEER procedure. The proposed standardized use of 3D TOE during TSP can help to ensure that all patients receive a consistently high level of assessment accuracy and reduce the variability and potential errors associated with the 2D TOE. This study has some limitations. First, despite including one hundred TSP attempts, the sample size may not be sufficient to capture potential outcomes in a broader patient population. This limitation suggests that further studies with larger cohorts are needed to confirm these results and better understand the impact of using 2D versus 3D measurements for septal puncture height. Second, MPR was always performed by the same operator, which limits the assessment of variability between operators, even though this may have led to more consistent data. However, this does not take into account the differences that may arise when multiple operators are involved. Third, we did not specifically analyse the time required during the procedure to carry out an MPR and whether this might have an impact on the overall procedure duration. On average, an experienced operator can carry out an MPR in about 30 s to 1 min. Based on our experience, the inclusion of MPR does not increase the duration of the procedure. On the contrary, by providing the operator with more detailed and precise anatomical information, MPR with improved clarity allows for more efficient decision-making, which can often translate into shorter procedure times.

## 5. Conclusions

The adoption of 3D TOE with MPR for the assessment of tenting height during TSP for mTEER may offer significant advantages over traditional 2D TOE. Enhanced visualization, increased accuracy, and potential for standardization make 3D TOE a superior method, likely improving procedural outcomes and patient safety. Therefore, integrating 3D TOE into routine practice for mTEER procedures should be strongly considered.

## Figures and Tables

**Table 1:** Baseline patient characteristics.

Variables	Total (*n* = 64)	Optimal 2D (*n* = 52)	Suboptimal 2D (*n* = 12)	*p* Value
Age, yrs	79 ± 8	79.10 ± 7.9	76.25 ± 9.9	0.442
Female, *n* (%)	25 (39.1)	22 (42.3)	3 (25)	0.338
BMI, kg/m^2^	25.4 ± 4.4	25.3 ± 4.8	26 ± 3	0.349
Hypertension, *n* (%)	48 (75)	41 (78.8)	7 (58.3)	0.156
Hyperlipidaemia, *n* (%)	37 (57.8)	29 (55.8)	8 (66.7)	0.537
Diabetes, *n* (%)	21 (32.8)	20 (38.5)	1 (8.3)	0.084
Previous MI, *n* (%)	20(31.3)	17 (32.7)	3 (25)	0.739
Previous PCI, *n* (%)	11 (17.2)	10 (19.2)	1 (8.3)	0.673
Previous CABG,	15 (23.4)	11 (21.2)	4 (33.3)	0.453
Previous TIA/stroke, *n* (%)	3 (4.7)	3 (5.8)	0 (0)	0.530
PAD, *n* (%)	7 (10.9)	4 (7.7)	3 (25)	0.115
COPD, *n* (%)	7 (10.9)	4 (7.7)	3 (25)	0.115
CKD (≥III), *n* (%)	21 (32.8)	18 (34)	3 (25)	0.736
Atrial fibrillation/atrial flutter, *n* (%)	37 (57.8)	31 (59.6)	6 (50)	0.747
History of cancer, *n* (%)	10 (15.6)	7 (13.5)	3 (25)	0.557
CRT-D, *n* (%)	6 (9.5)	3 (5.8)	3 (25)	0.060
NYHA ≥ III, *n* (%)	38 (58)	31 (59.6)	7 (58.3)	0.465
Previous valvular intervention, *n* (%)	5 (7.9)	3 (5.8)	2 (16.7)	0.105
Mechanical aortic valve *n* (%)	3 (4.7)	2 (3.8)	1 (8.3)	0.253
Previous TAVI *n* (%)	2 (3.1)	1 (1.9)	1 (8.3)	0.126
LVEF, %	50 (30.5–60)	50 (35.75–60)	50 (30.5–60)	0.809

Values are mean ± SD, median (interquartile range), or *n* (%). BMI: body mass index; MI: myocardial infarction; PCI: percutaneous coronary intervention; CABG: coronary artery bypass grafting; TIA: transient ischemic attack; PAD: peripheral artery disease; COPD: chronic obstructive pulmonary disease; CKD: chronic kidney disease; CRT-D: cardiac resynchronization therapy with defibrillator; NYHA: New York Heart Association; TAVI: transcatheter aortic valve implantation; LVEF: Left Ventricle Ejection Fraction.

**Table 2:** Baseline echocardiographic characteristics.

Variables (*n* = 64)	
PMR, *n* (%)	25 (39.1)
- Barlow disease (%) - FED (%)	16 (25) 9 (14.1)
SMR, N (%)	39 (60.9)
- Ischaemic, *n* (%) - Non-ischaemic, *n* (%)	24 (37.5) 15 (23.4)
EROA, cm^2^	0.45 (0.40–0.65)
VR, mL	63 (49–85)
VC, mm	7 (6–8)
VC3D, cm^2^	0.45 (0.35–0.7)
MVA, cm^2^	7 (5–7.75)
Mean transvalvular gradient, mmHg	2 (1–2)
Posterior leaflet length, mm	12 (10–15)
LVEF, %	50 (30.5–60)
LVEDD, mm	52 (47–59)
LVESD, mm	34 (28–42.5)
LVEDV, mL	127 (100.5–147)
LVESV, mL	54 (40–93)
PAPs, mmHg	45 (35–63)
TR (≥moderate), *n* (%)	40 (62.5)
TAPSE, mm	20 (17.25–23)
N. jets (>1), *n* (%)	25 (39.1)
LAVi, mL/mq	61 (49.5–73)

Values are median (interquartile range) or *n* (%). PMR: primary mitral regurgitation; SMR: secondary mitral regurgitation; EROA: effective regurgitant orifice area; VR: regurgitant volume; VC: vena contracta; VC3D: three-dimensional vena contracta area; MVA: mitral valve area; LVEF: left ventricular ejection fraction; LVEDD: left ventricular end-diastolic diameter; LVESD: left ventricular end-diastolic volume; LVESV: left ventricular end-systolic volume; PAPs: pulmonary artery systolic pressure; TR: tricuspid regurgitation; TAPSE: tricuspid annular plane systolic excursion; LAVi: left atrial volume index; LVEDV: left ventricular end-diastolic volume.

**Table 3:** Procedural characteristics and echocardiographic results.

Variables *n* = 64	
N. device	
1, *n* (%)	44 (68.8)
2, *n* (%)	19 (29.7)
3, *n* (%)	1 (1.6)
Fluoroscopy time, min	22 (15–28)
EROA, cm^2^	0.16 (0.12–0.21)
VR, mL	21 (16.25–27.5)
VC, mm	4 (2–5)
VC3D, cm^2^	0.18 (0.12–0.22)
MVA, cm^2^	3 (2–3.25)
Mean gradient, mmHg	3 (3–4)
LVEF, %	50 (30.5–60)
Pericardial effusion, *n* (%)	0 (0)
Cardiac perforation, *n* (%) Procedural success, *n* (%)	0 (0) 59 (92.2)

Values are mean ± SD, median (interquartile range), or *n* (%). EROA: effective regurgitant orifice area; VR: regurgitant volume; VC: vena contracta; VC3D: three-dimensional vena contracta area; MVA: mitral valve area; LVEF: left ventricular ejection fraction.

**Table 4:** Comparison between 2D and 3D assessments of TSP height.

	Method	Mean (SD)	CI 95%	Paired *t*-Test	Mean of Differences (CI 95%)
**TSP height (cm)**	MPR	4.59 (0.63)	4.46–4.71	0.0001	(0.16–0.30)
	2D	4.36 (0.61)	4.23–4.48		

*t*-test for paired data was used to assess the significance level. SD = standard deviation; CI = confidence interval. TSP = trans-septal puncture.

**Table 5:** Comparison between mean difference between 2D and 3D measurements of TSP height in optimal or suboptimal 2D views.

	Method	Median (IQR)	Mann–Whitney
**Mean difference between 2D and 3D measurements of TSP height (cm)**	Optimal 2D	0.2 (0.0–0.4)	0.0001
	Suboptimal 2D	0.5 (0.22–0.87)	

Mann–Whitney test was used to assess the significance level. IQR = interquartile range. TSP = trans-septal puncture.

## Data Availability

Data are available from the authors upon reasonable request.
